# Utilization of inpatient ophthalmology services in Taiwan—A nationwide population study

**DOI:** 10.1186/s12886-022-02765-5

**Published:** 2023-01-10

**Authors:** Chia-An Hsu, Min-Huei Hsu, Ju-Chuan Yen

**Affiliations:** 1 Department of Ophthalmology, Taipei Veterans General Hospital, Taipei, Taiwan; 2 Graduate Institute of Data Science, College of Management, Taipei Medical University, Taipei, Taiwan; 3 Department of Neurosurgery, Wan-Fang Hospital, Taipei Medical University, Taipei, Taiwan; 4 Department of Education and Research, Taipei City Hospital, Taipei, Taiwan; 5 Department of Ophthalmology, Ren-Ai Branch, Taipei City Hospital, Taipei, Taiwan; 6 Graduate Institute of Biomedical Informatics, College of Medical Science and Technology, Taipei Medical University, Taipei, Taiwan

**Keywords:** Ophthalmology, Inpatient utilization, Epidemiology

## Abstract

**Background:**

This study attempted to illustrate the demographic of inpatient eye careservice from 1997 to 2011 in Taiwan, and also the ophthalmic disease landscape and utilization change over time. These insights might apply to resource allocation planning and trainees’ better understandings of ophthalmic inpatient practice.

**Methods:**

This study utilized Taiwan’s National Health Insurance Research Database (NHIRD). Admission records of eye service that occurred since 1997 and until 2011 were included. Records were separated into operative and non-operative. The records were further divided according to their time: a group of early time before 2006 and a late one after 2006.

**Results:**

Patients’ mean age were 56 and 44 years for operative and non-operative records. The sex ratio (male to female) was 1.3, and the average of admission duration was 4 days. The average spending was around 1000 United State Dollars per admission and a gradually upgoing trend was also noted. The number of inpatient eye services decreased over time, from 3,248 to 2,174 in the studied period. Cases admitted for operation primarily underwent cataract surgery, vitrectomy, and scleral buckling during the studied period. Trabeculectomy emerged as another major indication of admission during the later time. Cases admitted for non-operative management were primarily corneal ulcer, glaucoma, and infection, including orbital cellulitis and lid abscess. Corneal ulcers made up a major proportion of admission records in the non-operative group during both periods.

**Conclusions:**

This study described the demographics of inpatient eye service in Taiwan. Ophthalmologist, especially trainees, and officials could make better policies according to the presented results in this study.

## Introduction

While most eye care services are delivered through outpatient department, the inpatient and emergency services still contribute significantly to the overall functionality of ophthalmology services. Emergent ophthalmic services play important role in treating urgent cases, such as trauma or acute angle closure glaucoma, in a timely manner to prevent vision loss. On the other hand, admitted ophthalmic patients receive care that mandate inpatient setting, such as operations that require general anesthesia or intraocular infections that warrant frequent intravitreous or intravenous antibiotics usage.

Previous literature demonstrated the landscape of primary ophthalmic inpatient admissions in the United States, Germany, Nigeria and Thailand [1–4]. This research attempted to probe and compare the difference between the landscape of admitted patients in those countries and Taiwan based on the Taiwanese National Health Insurance Research Database (NHIRD). Additionally, with our previous studies that examined outpatient and emergent eye care services of Taiwan [5, 6], we hope to complete the overall panorama of ophthalmology service in Taiwan. Hopefully, these insights might help guide policy decisions to improve resource allocation and enable better understanding of ophthalmic epidemiology. A preprint has previously been published [7].

## Materials and methods

This study was exempted by the Institutional Review Board of Taipei Medical University. Taiwan launched the single-payer National Health Insurance (NHI) to provide its citizens with universal healthcare on March 1, 1994, and the coverage rate has been over 99%. The NHIRD has been accessible for research purposes since 1996.

The NHIRD dataset used for this study includes the two-million-person sub-dataset for the years 1997–2011. This study analyzed two million beneficiaries with complete claims data from 1997 to 2011 who were randomly selected from among the larger NHIRD dataset. The rationale of choosing 2005 as the cutoff year is because that we planned to do the two-stage comparison, and why the earlier period was longer which was 9 years as the later period was 6 years was we had considered that ICD-9-CM had started to be used for reimbursement code from 2000 in Taiwan. And so, these two periods might be balanced based on ICD-9-CM codes. No significant differences in age, sex, or average-insured payroll-related premiums were identified between the randomly selected sample group and the larger population of all NHI enrollees.

We examined the utilization of ophthalmic inpatient services according to age, sex, disease, treatment, and cost using the randomly selected dataset. In addition, epidemiological factors were delineated. We analyzed the data according to two separate periods: the early period, from 1997 to 2005; and the late period, from 2006 to 2011. Data analyses were also performed for subgroups of surgical (operative) and non-surgical (non-operative) cases. Disease stratification was defined according to the International Classification of Diseases, Ninth Revision, Clinical Modification (ICD-9-CM) codes.

SAS 9.4, STATA15.0 and R 3.6.1 were used to perform descriptive and inferential statistics. Two sample t-tests were used to validate group differences, and p-values less than or equal to 0.05 were deemed significant.

## Results

This section may be divided by subheadings. It should provide a concise and precise description of the experimental results, their interpretation, as well as the experimental conclusions that can be drawn.

### Demographics of primary ophthalmic admissions

**Fig. 1:**
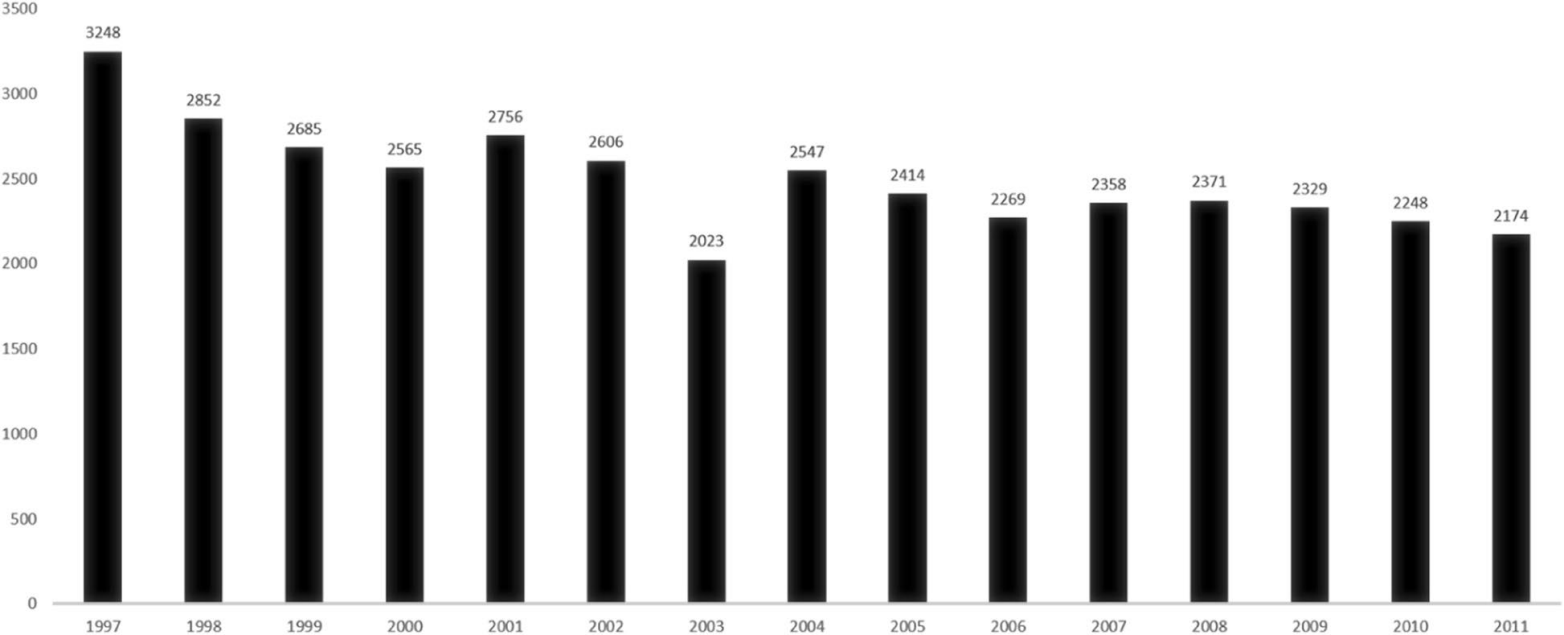
Yearly trends in ophthalmic admissions

**Fig. 2:**
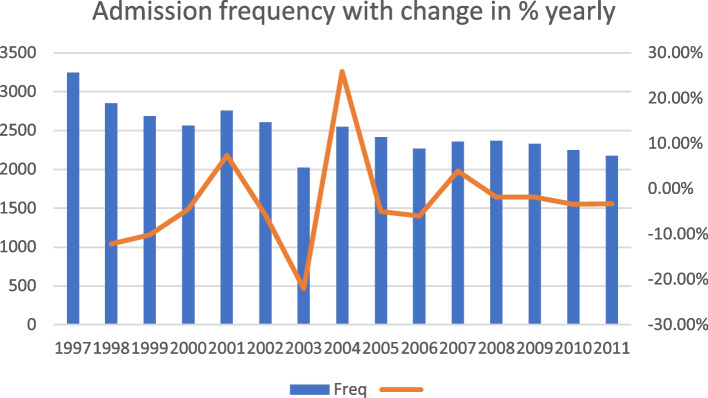
Yearly change in % of ophthalmic admissions

The yearly trends in ophthalmic admissions are shown in Fig. 1, which revealed decreasing trend in ophthalmic admissions over time. The number of admissions decreased from 3,248 to 1997 to 2,174 in 2011. Notably, only 2023 admissions were recorded in the year 2003. While Fig. 2 demonstrated change in percentage during these two periods.

A total of 23,696 admissions were recorded for the early period (1997–2005), with a mean of 2,632.9 and a standard deviation of 331.0 admissions per year. By contrast, 13,749 total admissions were recorded for the late period (2006–2011), with a mean of 2291.5 and a standard deviation of 75.3 admissions per year. A significantly reduced frequency of admissions frequency was observed for the late period (*p* = 0.015) compared with the early period. The mean age of ophthalmic admission patients was 56.3 years, with a standard deviation of 22.2 years during the early period, whereas the mean age was significantly lower for the late period at 55.4 years, with a standard deviation of 20.9 years. The male-to-female ratio was 1.333 to 1 during the early period and 1.285 to 1 during the late period, which represented a significant difference (*p* < 0.001) that was associated with a reduction in the male predominance associated with admissions during the late period. The mean costs of admission were 956 USD (United States Dollar) during the early period and 1028 USD during the late period, with standard deviations of 589 USD and 635 USD, respectively, indicating a significant increase in costs during the late period (*p* < 0.001). The mean hospital stay was 4.0 days, with a standard deviation of 4.7 days, for the early period, compared with a mean of 3.6 days, with a standard deviation of 4.0 days, for the late period, indicating a significantly shorter hospital stay during the late period (*p* < 0.001, Table 1).

**Table 1:** Demographics of Primary ophthalmic admission in Taiwan

	Early(1997–2005, *n* = 23,696)		Late(2006–2011, *n* = 13,749)		*p*-Value
	Mean	SD	Mean	SD	
**Number of admissions**	2632.89	331.06	2291.5	75.3	0.015*
**Age**	56.26	22.22	55.39	20.87	< 0.01*
	**Male**		**Male**		
**Sex**	13,537(57.1%)		7734(56.3%)		0.101+
	**Mean**	**SD**	**Mean**	**SD**	
**Cost (United State Dollar)**	956.1	589.4	1027.7	635.7	< 0.01*
**Hospital Stay (Days)**	4.039922	4.717708	3.599462	3.9514	< 0.01*

^*^Two-sample t-test

^+^Chi-square test

### Comparison between operative and non-operative admissions

We classified diseases codes as operative and non-operative, based on whether the patient received surgery (Table 2).

A total of 34,046 admissions were recorded for operative purposes, with a mean frequency of 2,270 and a standard deviation of 283 admissions each year, compared with 3,399 nonoperative cases, with a mean frequency of 227 and a standard deviation of 42 admissions per year. The mean age was 56.7 years, with a standard deviation of 21.6 years, among patients admitted for operative purposes, compared with the significantly younger mean age of 48.4 years with a standard deviation of 22.1 years observed among patients admitted for non-operative purposes (*p* < 0.001). The male-to-female ratio of patients undergoing operative treatment was 1.320 to 1, which did not differ significantly from the ratio of 1.266 to 1 for non-operative treatment (*p* = 0.255). The mean cost of 1039 USD with a standard deviation of 596 USD was significantly higher for patients receiving operative treatment compared with the mean cost of 412 USD with a standard deviation of 393 USD for patients receiving non-operative treatment (*p* < 0.001). The mean length of hospital stay was 3.7 days, with a standard deviation of 4.3 days, for operative cases, which was significantly shorter than the mean length of hospital stay for non-operative cases of 5.6 days with a standard deviation of 5.6 days (*p* < 0.001).

**Table 2:** Comparisons of operative and non-operative admissions

	Operative (*n* = 34,046)		Non-operative (*n* = 3399)		*P*-Value
	Mean	SD	Mean	SD	
**Number of admissions**	2269.7	283.2	226.6	42.1	
**Age**	56.6	21.6	48.4	22.1	< 0.01*
**Sex**	**Male**	**Female**	**Male**	**Female**	
	19,372	14,674	1899	1500	0.255+
	**Mean**	**SD**	**Mean**	**SD**	
**Cost (United State Dollar)**	1039.3	596.0	412.1	393.1	< 0.01*
**Hospital Stay (Days)**	3.7	4.3	5.6	5.6	< 0.01*

*Two-sample t-test

+Chi-square test

Among non-operative cases during the early period, the five most common diagnoses were corneal ulcer (23.71%), eyeball contusion (4.65%), acute angle-closure glaucoma (3.22%), orbital cellulitis (3.13%), and unspecified glaucoma (2.22%; Table 3). The five most common non-operative diagnoses during the late period were corneal ulcer (24.45%), orbital cellulitis (6.09%), optic neuritis (4.00%), vitreous hemorrhage (3.00%), and eyelid abscess (2.73%; Table 3).

**Table 3:** Five most frequent non-operative diagnoses codes

Early (1997–2005, *n* = 2299)			Late (2006–2011, *n* = 1100)		
Diagnosis	Count (*n*)	Percentage	Diagnosis	Count (*n*)	Percentage
**Corneal ulcer**	545	23.7%	**Corneal ulcer**	269	24.5%
**Contusion of eyeball**	107	4.6%	**Orbital cellulitis**	67	6.1%
**Acute angle-closure glaucoma**	74	3.2%	**Optic neuritis**	44	4.0%
**Orbital cellulitis**	72	3.1%	**Vitreous hemorrhage**	33	3.0%
**Unspecified glaucoma**	51	2.2%	**Abscess of eyelid**	30	2.7%

The five most common diagnoses for operative cases during the early period were phacoemulsification (19.01%), other extracapsular extraction of the lens (14.42%), other mechanical vitrectomy (10.59%), extracapsular extraction of the lens by simple aspiration technique (6.07%), and other scleral buckling (5.06%), whereas phacoemulsification and aspiration of cataract (25.67%), other mechanical vitrectomy (22.68%), other extracapsular extraction of the lens (6.2%), other scleral buckling (4.43%), and trabeculectomy ab externo (4.27%) were the leading five diagnoses during the late period (Table 4).

**Table 4:** Five most frequent operative diagnoses codes

Early (1997–2005, *n* = 21,397)			Late (2006–2011, *n* = 12,649)		
Diagnosis	Count (*n*)	Percentage	Diagnosis	Count(*n*)	Percentage
**Phacoemulsification and aspiration of cataract**	4067	19.0%	**Phacoemulsification and aspiration of cataract**	3247	25.7%
**Other extracapsular extraction of lens**	3085	14.4%	**Other mechanical vitrectomy**	2869	22.7%
**Other mechanical vitrectomy**	2267	10.6%	**Other extracapsular extraction of lens**	786	6.2%
**Extracapsular extraction of lens by simple aspiration (and irrigation) technique**	1298	6.1%	**Other scleral buckling**	560	4.4%
**Other scleral buckling**	1082	5.1%	**Trabeculectomy ab externo**	540	4.3%

During the early period, from 1997 to 2005, cataract surgery was a common reason for operative cases, followed by vitrectomy and scleral buckling. By comparison, during the late period, from 2006 to 2011, cataract surgery remained a primary reason for operative cases, followed by vitrectomy, scleral buckling, and trabeculectomy.

## Discussion

### Demographics of primary ophthalmic admissions

In the studied population, the number of annual admissions ranged from 3,248 to 1997 to 2,174 in 2011. A decreasing trend of the number of annual admissions was noted. The decreasing tend was likely related to the tendency of performing cataract surgery at clinic instead of inpatient department over time.

The number of ophthalmic admissions for the year 2003 was only 2,023. Similar to how the current coronavirus disease 2019 (COVID-19) pandemic has overwhelmed healthcare since 2020, in 2003, the SARS epidemic occurred in Taiwan. The pandemic impacted clinical management, which was reflected in the changes in ophthalmology admission services, many of which are considered elective procedures.

The annual ophthalmic admission rate of Taiwan was reported as 108–162 per 100,000 population, which was higher than that of 16 per 100,000 population in the United States [1]. The mean cost of ophthalmic admissions was approximately 1,000 USD in Taiwan, which was one-ninth the average cost of 9,700 USD in the United States [1]. The mean age for the ophthalmic patients admitted in Taiwan was approximately 56 years, which was much older than the mean age of 44 years reported for the United States [1]. This discrepancy may be due to the fact that cataract surgery still represented a significant proportion of ophthalmic admissions in Taiwan while the same operation was mainly provided under an outpatient setting in the United States.

The male-to-female ratios revealed a male predominance in both countries, with men representing approximately 56.5% of admissions in Taiwan compared to that of 51.6% in the United States [1]. Another study from Nigeria also demonstrated a similar male-to-female ratio of 1.30 to 1 [2]. The mean hospital stays associated with Taiwanese ophthalmic admissions were slightly longer than that of the United States. Patients spend an average of 3.7 days for operative and 5.6 days for non-operative diagnoses in hospital, compared to that of 3.2 days for both types of cases in the United States [1]. The average hospital stay in Nigeria was even shorter, at 2.86 days [2]. The length of the hospital stay in Germany was similar to that reported for Taiwan. As for the trend of Similar to Germany^6^, the hospital stay showed a declining trend from 3.9 to 3.4 days.

### Admissions for operative diagnoses

Admissions for operative diagnoses outnumbered that of non-operative diagnoses, at a ratio of 10 to 1, indicating that ophthalmic admissions were primarily utilized for surgical purposes. The most common underlying reasons for ophthalmic admission in our study included cataract surgery during both the early (39.50%) and late periods (31.89%), followed by vitrectomy (10.59% in the early period and 22.68% in the late period), scleral buckling (5.06% in the early period and 4.43% in the late period), and trabeculectomy (4.27%), which emerged during the late period. Cataract surgery played a pivotal role in ophthalmic admissions but demonstrated a declining trend, similar to the study performed in Germany [3]. By contrast, vitrectomy showed an upward trend in ophthalmic admissions, similar to the studies performed in the United States [1] and Germany [3], whereas scleral buckling revealed a downward trend, which might be due to advancements in vitrectomy technology. Due to an aging population and the complexity of glaucoma surgery, trabeculectomy began to emerge as a common procedure during the late period. In Nigeria [2] and Thailand [4], the primary reasons for inpatient ophthalmic services were cataract and glaucoma surgeries, which reflect an aging population and were similar to the Taiwanese ophthalmic admission landscape observed in this study. The study from Thailand also showed that the five most common inpatient eye diseases, based on ICD-10-CM codes, were disorders of the lens; conjunctivitis; disorders of the sclera, cornea, iris, and ciliary body; disorders of the choroid and retina; and glaucoma [4].

As Table 5 showed Taiwan’s inpatient admission utilizations might be an epitome for the admitted disease landscape overtime globally, which demonstrated decreasing cataract surgery performed as inpatient services and increased vitreoretinal surgery as well as trabeculectomy of glaucoma patients, and also the downward trending of the hosptial stay and uprising costs worldwide.

**Table 5:** .

	Taiwan	USA	Nigeria	Germany	Thailand
Costs USD	1000	9700			
Ophthalmic admission	108–162/100,000	16/100,000			
Mean age	56 y/o	44 y/o			
M/F + F %	56.5%	51.5%			
Hospital stay days	3.7 VS 5.6	3.2	2.86	3.9–3.4	
Operative	Cataract surgery topped				
Vitrectomy uptrending	+	+		+	
Scleral Buckling downtrending	+			+	
Trabeculectomy uptrending	+		+		
Infection uptrending	+	+			+
Cataract surgery downtrending	+			+	

### Admissions for non-operative diagnoses

Among non-operative diagnoses, corneal ulcers (from 23.71 to 24.45%) were the most common during both the early and late periods, followed by contusions of the eyeball (4.65% in the early period), orbital cellulitis (3.13%), acute angle-close glaucoma (3.22%), and unspecified glaucoma (2.22%) during the early period; whereas orbital cellulitis (6.09%), optic neuritis (4.0%), vitreous hemorrhage (3.0%), and abscess of the eyelids (2.73%) were more common during the late period. Corneal ulcer has remained the leading cause of admissions for both the early and late periods, accounting for almost a quarter of non-operative ophthalmic admissions. However, an upward trend in other infections, such as orbital cellulitis (from 3.13 to 6.09%) and lid abscesses (2.22%), emerged during the late period. Similarly, the most common diagnosis for ophthalmic admissions was orbital cellulitis, followed by orbital fracture and lid abscesses in the United States [1]. The emergence of methicillin-resistant *Staphylococcus aureus *[8] during the last decade in both Taiwan and the United States has been associated with the increased incidence of orbital and lid infections. In another study performed at a Nigerian tertiary hospital [2], the primary non-operative cause for ophthalmic admission was ocular trauma, which accounted for 14.3% of ophthalmic admission; although the prevalence of ocular trauma in our study was only 4.65%, this propensity was reflected during the early period. Another common reason for non-operative admissions during the early period was glaucoma, especially angle-closure glaucoma; however, this diagnosis was more common during the early period. The reasons for this shift might be due to the high prevalence of primary angle-closure glaucoma (PACG) among people from Asia, especially those from Taiwan, and the laser treatment that has been developed for PACG prevention can be performed as an outpatient eye care service, which may have decreased ophthalmic admissions for PACG.

The most distinct feature in our study was that corneal ulcers were the top diagnoses in our present study for both the early and late periods. In Taiwan, access to contact lenses is available without an ophthalmologist’s prescription, despite regulations that aim to require ophthalmologist recommendations. Unfortunately, corneal ulcers can develop due to poor education regarding the appropriate use and hygiene of contact lenses for the users. Keratitis accounted for roughly one million outpatient and emergency eye care visits in the United States [9]. Keratitis is known to be a potential precursor of corneal ulcers. Corneal ulcers frequently occur among contact lens wearers, especially those who use extended contact lenses. One study, performed as a retrospective chart review, revealed that corneal ulcers were the most prevalent among young women aged 25 to 34 years (with an incidence rate of 60.3 per 100,000 person-years) [10]. Corneal ulcers can lead to poor clinical outcomes, such as corneal scarring or blindness; therefore, our results raise concerns regarding the appropriate use of prescriptions of contact lenses in Taiwan, which might necessitate additional regulations. Another factor contributing to the high rate of corneal ulcers might be the relatively high prevalence of myopia [11] and the relevant orthokeratology of rigid, gas-permeable, extended-wear contact lenses for myopia control that are used by the grade school and high school student populations in Taiwan.

The mean ages of operative and non-operative patients were 56 and 48 years, respectively, indicating that non-operative patients were much younger than operative patients. Operative patients were primarily treated for cataracts and vitreoretinal or glaucoma surgeries, which are diagnoses primarily associated with aging; by contrast, the non-operative patients were primarily treated for infection, trauma, and inflammation (corneal ulcer, orbital cellulitis, and blunt eye injuries), which are diagnoses associated with younger demographics [10].

## Conclusion

The landscape of primary ophthalmic admissions in Taiwan were probed and summarized in this study. The downward trending, incresasing costs, with younger age presentation of ophthalmic admissions were revealed in the present study. Furthermore, cataract surgery topped in both periods, yet the trend of dereasing cataract surgery and scleral buckling with increasing vitreoretinal and glaucoma surgery were showed in the operative cases. Corneal ulcer cases also topped in both periods and infection cases such as orbital cellulitis and lid abscess as well as optic neuritis and vitreous hemorrhage trending upward in the non-operative cases. This study provides the necessary information for future policy makers and ophthalmologists to understand the completed panorama of Taiwan’s eye care services and make informed decisions. The resource allocations should be more focused retinal, glaucoma, infection controlling and prevention of cornea, lid and orbit as the global trend of aging population and ophthalmic surgical technology advancement.

## Data Availability

The specific dataset used to conduct this study is not publicly available due to the management policy of National Health Insurance Research Database. Data from the National Health Insurance Research Database, now managed by the Health and Welfare Data Science Center (HWDC), can be obtained by interested researchers through a formal application process addressed to the HWDC, Department of Statistics, Ministry of Health and Welfare, Taiwan (https://dep.mohw.gov.tw/DOS/lp-2506-113.html, accessed on 1 May 2022).
